# Semi-supervised few-shot learning approach for plant diseases recognition

**DOI:** 10.1186/s13007-021-00770-1

**Published:** 2021-06-27

**Authors:** Yang Li, Xuewei Chao

**Affiliations:** 1 College of Mechanical and Electrical Engineering, Shihezi University, Xinjiang, China; 2 School of Electrical and Information Engineering, Tianjin University, Tianjin, China

**Keywords:** Classification, Transfer learning, Self-adaption, Deep learning

## Abstract

**Background:**

Learning from a few samples to automatically recognize the plant leaf diseases is an attractive and promising study to protect the agricultural yield and quality. The existing few-shot classification studies in agriculture are mainly based on supervised learning schemes, ignoring unlabeled data's helpful information.

**Methods:**

In this paper, we proposed a semi-supervised few-shot learning approach to solve the plant leaf diseases recognition. Specifically, the public PlantVillage dataset is used and split into the source domain and target domain. Extensive comparison experiments considering the domain split and few-shot parameters (N-way, k-shot) were carried out to validate the correctness and generalization of proposed semi-supervised few-shot methods. In terms of selecting pseudo-labeled samples in the semi-supervised process, we adopted the confidence interval to determine the number of unlabeled samples for pseudo-labelling adaptively.

**Results:**

The average improvement by the single semi-supervised method is 2.8%, and that by the iterative semi-supervised method is 4.6%.

**Conclusions:**

The proposed methods can outperform other related works with fewer labeled training data.

## Background

In agricultural production, the monitoring of plant growth and health status is helpful to guide farmers to timely take suitable measures to guarantee the yield and quality [1]. In practice, the recognition of plant diseases mainly depends on farmers' experience in many countries and areas, often by observing changes in plant leaves' appearance. With the development of digital agriculture and precision agriculture, it is necessary to bring the computer and sensing techniques [2–4] into the traditional agricultural production to achieve efficient and automatic production. Thus, the automatic recognition of plant leaf diseases is essential and related to other agricultural researches [5–7].

At present, the methods to classify plant leaf diseases are mainly through the analysis of plant leaf images, e.g., RGB images, near-infrared images, and hyperspectral images [8–10], taken by general cameras or unmanned aerial vehicle (UAV). The deep learning technique is a powerful tool in the image analysis process, which has achieved excellent performances in many areas, such as leaf segmentation [11], leaf spot detection [12], leaf diseases classification [13–17], and others [18, 19]. The deep learning models in these studies all have many layers, in which there are a large number of parameters to train.

Although this typical deep learning method to train models from large-scale datasets indeed has achieved good performance, the community has begun to rethink this learning approach at a crossroad. One way is to go deeper, with more complex networks and larger datasets. For example, many works focused on collecting a big dataset at a high cost [20], designing a deeper model with optimization [21], designing the ensemble model [22], etc. The other way is to solve the classification problem with few data, also called few-shot learning, which is more suitable for practical applications. For example, some other works focused on model compression by pruning [23], shallow model [24], and lightweight network [25].

From our point of view, we are in favour of the few-shot learning approach. There are two reasons for that. First, it is hard and high-cost to collect big scale dataset for all the problems in agriculture. Some plant diseases may be so rare that collecting large numbers of samples is impractical. The annotation and identification of collected samples also require experts or experienced farmers' efforts, so massive data annotation is time-consuming and laborious. Second, the deep learning model has a deep network structure and massive parameters, requiring higher and more hardware resources to train and test. Moreover, the deployment of deep learning models on the portable terminals is difficult. Learning from few data with a small model to classify is a meaningful and promising study in practical applications due to the low cost of data.

The related studies on few-shot classification in the agricultural field are still relatively few at this beginning stage, but it has received increasing attention. A handful of research has emerged, focusing on the few-shot classification in agriculture [26–30]. Specifically, Hu et al. used the data augmentation to solve few-shot classification of tea leaf diseases based on the generative adversarial network [26]. Argüeso et al. used the transfer learning method to transfer knowledge from the source domain to the target domain, and the testing accuracy was above 90% under 6-way and 80-shot [27]. Li et al. used the triplet loss to train feature extractor based on distance metric comparison and focused on combining few-shot algorithms and terminal realization [28]. Zhong et al. used the conditional adversarial autoencoders to generate samples for the zero-shot and few-shot diseases recognition based on the visual and semantic features [29]. Li et al. used the metric learning to analyze the single domain and cross domain of crop pests and plant diseases recognition [30]. The above studies are all based on the supervised learning schemes, using only a few labeled samples and ignoring the helpful information of unlabeled samples. Note that, in many application scenarios, the unlabeled samples may be easier to collect. In other words, in addition to a few labeled samples, there may also be many unlabeled samples, so how to make full use of the unlabeled data is indeed a significant and meaningful issue.

In this paper, we proposed a semi-supervised few-shot classification method based on transfer learning. The semi-supervised method uses both a few labeled samples and many unlabeled samples to train a model. Extensive experiments were carried out on the public dataset PlantVillage and compared with the Ref [27], which was also based on transfer learning. The transfer learning technique needs to split the dataset into source domain and target domain. The reference only considered one domain split situation; we further compared three more domain splits to validate the proposed method's correctness and generalization. Besides the domain split, we also considered other influencing factors, such as few-shot parameters and semi-supervised iteration.

The contributions of this work can be summarized as three-fold:

1. We carried out the first semi-supervised few-shot work in the field of plant leaf disease recognition.
2. We proposed to use the confidence interval to select unlabeled samples for pseudo-labeling in the semi-supervised process adaptively.
3. We considered many factors to verify the proposed method's correctness and generalization, including the domain split, few-shot parameters, and semi-supervised iteration.

## Materials

PlantVillage is a public dataset with 38 classes of plant leaf diseases and healthy crops. The number of samples in each category is not equal. The Ref. [31] augmented the images for those classes with fewer samples. In this shared dataset, the minimum number of samples per class is 1000, and the maximum number is 5507, corresponding to the orange citrus greening disease. To avoid the impact of unbalanced data distribution, we randomly select 1000 images per category to assemble the used balanced dataset. All the images are resized to 84*84*3, corresponding to the RGB channels.

The dataset was split into a source domain and a target domain, without intersections between these two parts. The main reason is the inherent limitations of few data, which cannot provide enough information of categories. Mimicking the way humans learn, we hope the few-shot model can learn new tasks from few samples based on basic knowledge and past experiences. The target domain only provides a few labeled data to train the model, then it is hoped to generalize to previously unseen samples. In fact, this way of learning is similar but different with the typical transfer learning, which generally has large number of training data in the source domain.

**Fig. 1:**
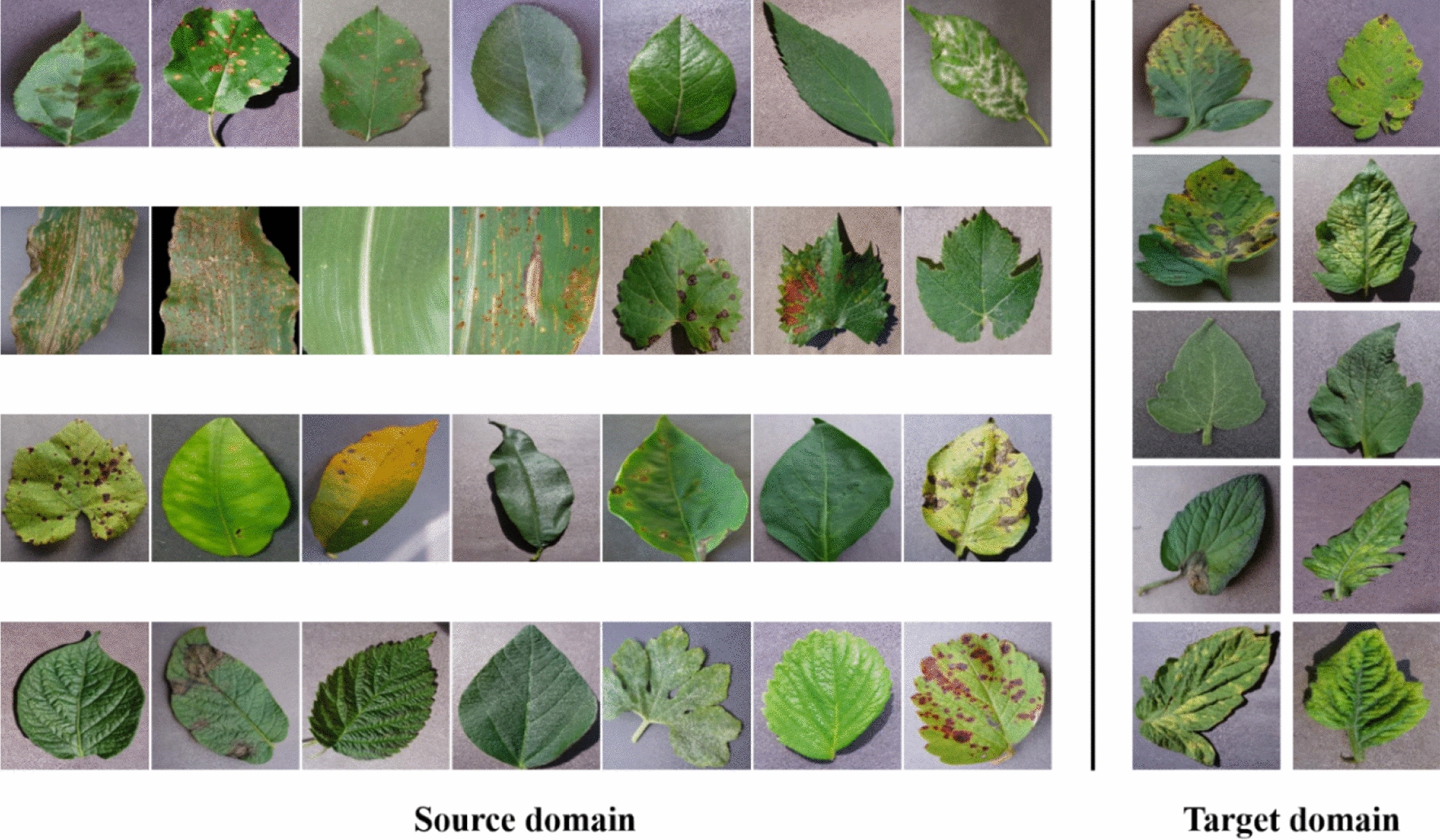
Some examples corresponding to the Split-1

Generally, different splits of the source domain and target domain in the same dataset will lead to different difficulties during the transfer learning process because the fitness of transferred knowledge between the source domain and target domain is different. To verify the correctness and generalization of the proposed semi-supervised few-shot classification of plant leaf diseases, we performed three different splits of the source domain and target domain on the PlantVillage dataset. The split details are shown in Table 1, specifically, some image examples corresponding to the Split-1 are shown in Fig. 1.

**Table 1:** The split modes of source and target domain

Split Mode	Source (28 classes in total)	Target (10 classes in total)
	Crop (number of categories)	Crop (number of categories)
Split-1	Apple(4), Blueberry(1), Cherry(2), Corn(4), Grape(4), Orange(1), Peach(2), Pepper(2), Potato(3), Raspberry(1), Soybean(1), Squash(1), Strawberry(2)	Tomato(10)
Split-2	Blueberry(1), Corn(4), Orange(1), Peach(2), Pepper(2), Potato(3), Raspberry(1), Soybean(1), Squash(1), Strawberry(2), Tomato(10)	Apple(4), Cherry(2), Grape(4)
Split-3	Apple(4), Blueberry(1), Cherry(2), Orange(1), Pepper(2), Potato(3), Raspberry(1), Soybean(1), Squash(1), Strawberry(2), Tomato(10)	Corn(4), Grape(4), Peach(2)

In Table 1, for all the three split modes, the number of classes in the source domain is 28, and that in the target domain is 10. For the sake of presentation, specific disease names are not expanded, and only the number of diseases for each crop is listed. For example, Apple(4) refers to the four categories corresponding to Apple: apple scab, black rot, cedar apple rust, and healthy.

## Methods

### Transfer-based few-shot classification

#### Overall framework

**Fig. 2:**
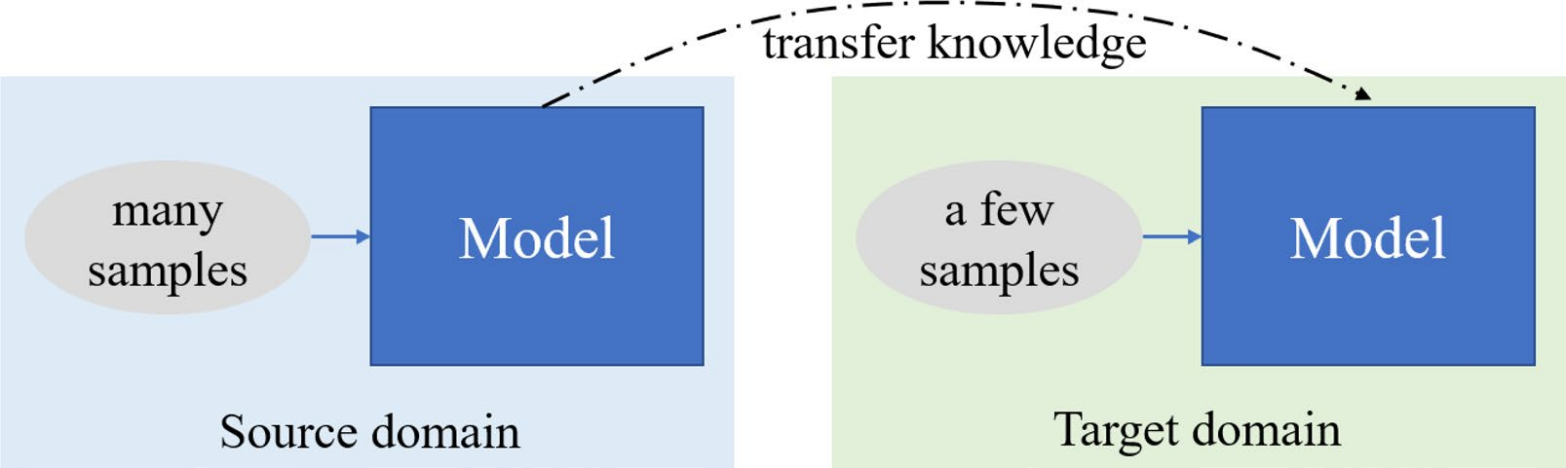
The overall framework of transfer-based few-shot classification

The overall framework of typical few-shot classification based on transfer learning is shown in Fig. 2. As known, the used dataset was split into a source domain and a target domain. It is given that there are many available labeled data in the source domain to train the model to learn basic knowledge and then transfer the trained network with parameters to the target domain as transferred knowledge. In the target domain, there are only a few labeled data can be used to updated the model, called fine-tuning. Due to the small number of provided labeled samples, this kind of problem is called few-shot classification.

#### Model structure

**Fig. 3:**
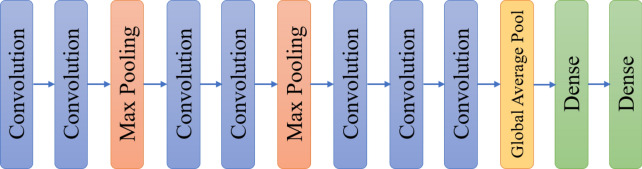
The structure of the used model

The model in this work is designed based on a convolutional neural network (CNN), which has been widely used in image processing [32–35]. The structure of the used model is shown in Fig. 3.

As seen in Fig. 2, the model in the source domain has same structure as that in the target domain. Given the size of input images, each layer's details in the model are shown in Table 2. According to the fine-tuning setting in transfer learning, the parameters in the first several layers are fixed and non-trainable, those in the last dense layers are trainable. The pooling layers have no parameters.

**Table 2:** The details of each layer in the model

Layers	Output size	Parameters	Fine-tuning
Input	(84, 84, 3)	0	–
Convolution	(84, 84, 64)	1792	Non-trainable
Convolution	(84, 84, 64)	36,928	Non-trainable
Max pooling	(42, 42, 64)	0	–
Convolution	(42, 42, 128)	73,856	Non-trainable
Convolution	(42, 42, 128)	147,584	Non-trainable
Max pooling	(21, 21, 128)	0	–
Convolution	(21, 21, 256)	295,168	Non-trainable
Convolution	(21, 21, 256)	590,080	Non-trainable
Convolution	(21, 21, 256)	590,080	Non-trainable
Global average pool	(256)	0	–
Dense	(128)	32,896	Trainable
Dense	(N)	128*N + N	Trainable

In Table 2, there are seven convolution layers and three pool layers. For the first two convolution layers, the number of filters is 64, and the padding mode is the same padding. For the max-pooling layer, the purpose is to halve the space size and maintain the same number of channels. The situation for the following convolution layers is similar, and the number of filters is 128 and 256, respectively.

Note that the last dense layer refers to the softmax classifier. Thus, the number of its output neurons should be the same as the number of categories classified, here written as N. For the source domain, the training samples from all 28 classes are used, so the N is 28. But for the target domain, the N is variable.

The few-shot classification in this paper uses the typical definition: N-way k-shot. That means there are N categories and k samples per category available to fine-tune the transferred model, which is wished to distinguish these N classes in the target domain. As described, there are in total ten classes in the target domain. Hence, the N can be anyone smaller than 10, generally equal to 3 or 5.

#### Training, fine-tuning, and testing

The training stage occurs in the source domain with a batch size of 16. The Adam optimizer with its default parameters is adopted, and the categorical cross-entropy is used as the loss function. There are 20% data in the source domain split as the validation set to check the model’s training status.

The fine-tuning stage occurs in the target domain, where only provides a few labeled data. The model trained in the source domain will be transferred to the target domain. The number of neurons in its last dense layer is replaced with N. If all the model parameters are fine-tuned, the serious overfitting problem is inevitable because of the small number of labeled training data. Hence, as shown in Table 2, only the parameters in the last two dense layers are fine-tuned and trainable, while the parameters in other layers are fixed.

The testing stage also occurs in the target domain, based on the fine-tuned model. The few-shot classification problem definition is the N-way k-shot, and the N classes are randomly selected from the ten classes in the target domain. The selected categories to be classified may be similar or significantly different, resulting in different task difficulty. So, only one experiment is not enough. We performed ten times for each group (N-way k-shot) of experiments, and then the average accuracy is output.

### Semi-supervised few-shot classification

In “Transfer-based few-shot classification” section, the few-shot classification based on transfer learning was introduced, which includes two main parts. One is the source domain to learn basic knowledge from a large number of labeled data. The other is the target domain to fine-tune parts of the transferred network from a few labeled data to adapt the specific classification tasks. Our essential contribution is to propose the semi-supervised few-shot classification method, working in the target domain.

#### Single semi-supervised few-shot classification

**Fig. 4:**
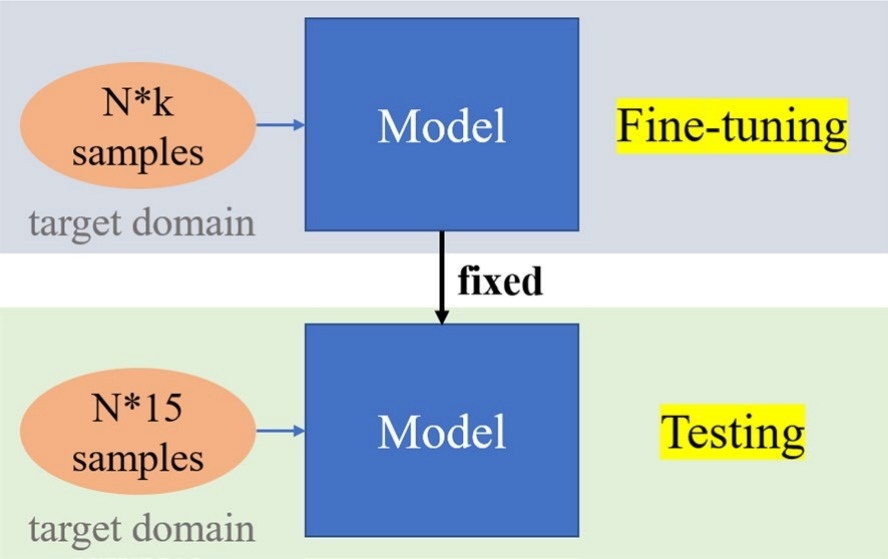
The typical fine-tuning and testing process

As comparison, the typical fine-tuning and testing process of few-shot classification is shown in Fig. 4.

Based on the N-way k-shot definition in “Model structure” section, the N*k samples are used to update the last two dense layers' parameters, and then the fine-tuned model is fixed. We randomly select 15 samples per category, total N*15 samples, to test the fixed model's few-shot performance, referring to Ref [36, 37]. The testing process will be performed ten times to obtain the average few-shot accuracy in this work.

**Fig. 5:**
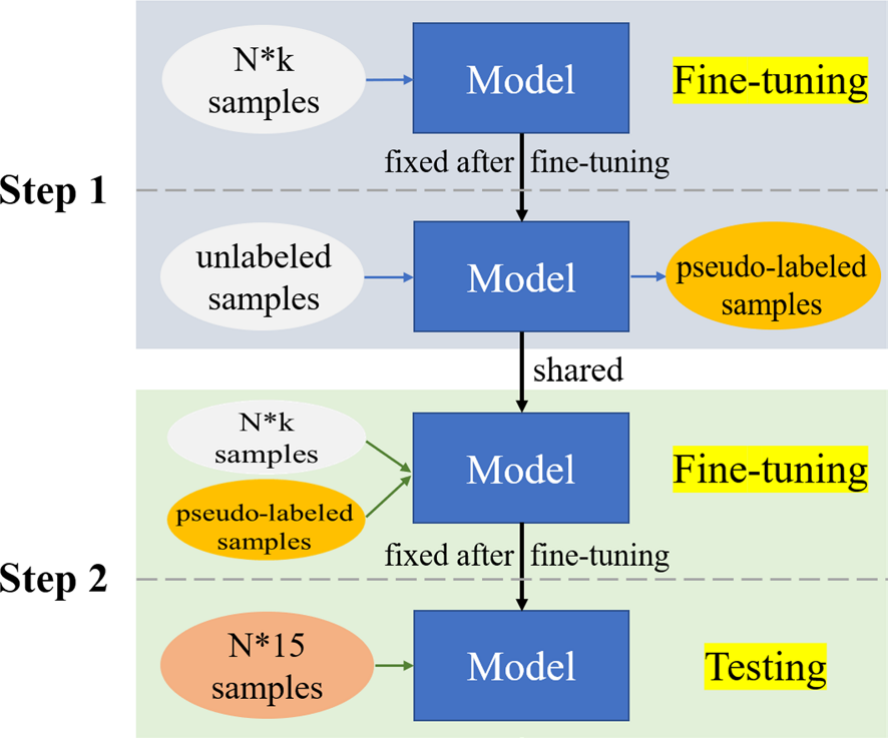
The single semi-supervised few-shot method

To improve the few-shot classification performance, we propose the semi-supervised method, shown in Fig. 5. It is shown that there are two steps to complete.

In step 1, the N*k samples with true labels are used to fine-tune the transferred model and then fix all the parameters after fine-tuning. All the unlabeled samples are then fed to the fixed model to make predictions and select some of them as pseudo-labeled samples. The pseudo-label means the prediction label given to the sample through the judgment of the model.

In step 2, both the N*k labeled samples and the selected pseudo-labeled samples from step 1 are used to fine-tune the model again. The trainable parameters are still those in the last two dense layers. After the fine-tuning, the model is fixed again and tested on the N*15 samples. Because the pseudo-labeled samples in the semi-supervised methods can be selected once or more times, the above-described process is also called single semi-supervised few-shot classification.

#### Iterative semi-supervised few-shot classification

**Fig. 6:**
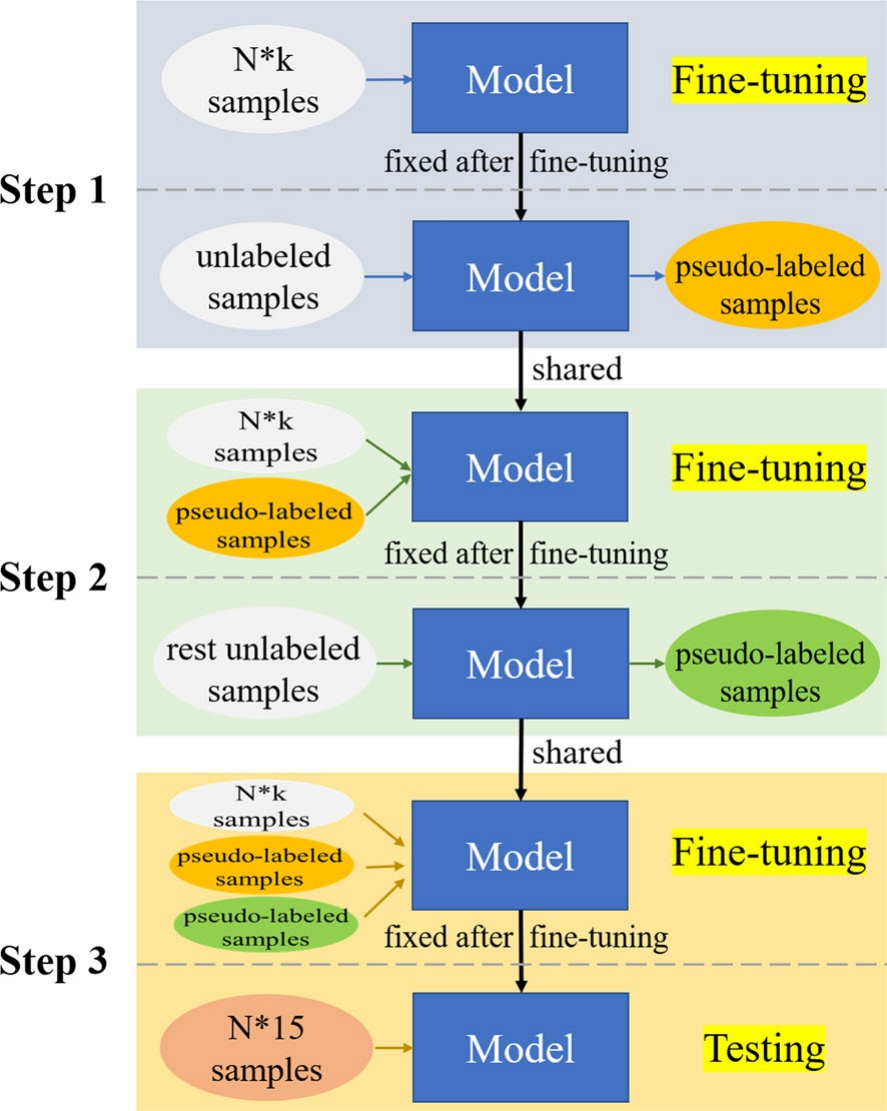
The iterative semi-supervised few-shot method

Based on the single semi-supervised few-shot classification, we further propose the iterative semi-supervised few-shot classification, shown as Fig. 6.

The iterative semi-supervised few-shot method has three steps, but the whole operation is similar to the single semi-supervised method. The difference is that the pseudo-labeled samples are selected twice. In particular, step 1 of the iterative semi-supervised method is the same as that of the single semi-supervised method. After the fine-tuning stage in step 2, the rest unlabeled samples except the selected pseudo-labeled samples in step 1 are fed to the fixed model to select pseudo-labeled samples again. In step 3, the N*k labeled samples and the pseudo-labeled samples from both steps 1 and 2 are used together to fine-tune the parameters in the last two dense layers of the model, and then the model is fixed finally to do the testing on the N*15 samples.

#### Adaptive selection of pseudo-labeled samples

In Figs. 5 and 6, the unlabeled samples are fed to the model to select some of them for pseudo-labeling. But how to determine the number of pseudo-labeled samples? The simplest method is to set some number, e.g., 5 or 10, manually. However, this is not a wise choice, owing to the low fitness for different tasks.

It should be noted that the selection of pseudo-labeled samples is a double-edged sword. If we can get many suitable pseudo-labeled samples in the semi-supervised process, the shortage of few data with original labels can be solved. But if we get many unsuitable pseudo-labeled samples, e.g., with many wrong labels, there will be terrible impacts on the few-shot performance due to the misdirection of fine-tuning by the wrong labels. Besides, if we are too careful to select only a few pseudo-labeled samples, the improvement will also be tiny.

We propose an adaptive selection method based on the confidence interval to solve the pseudo-labeled samples' selection problem. Specifically, only when the prediction confidence is larger than 99.5% will the unlabeled sample be selected by the model to pseudo-label as the predicted category. Although these given labels are called pseudo-labels, they should be almost entirely consistent with the real ones, as the model has such high confidence in the predictions. In this case, the model will adaptively determine the number of pseudo-labeled samples under different experiments.

## Results

This section carried out the comparison experiment with other related work and further experiments considering the factors of domain split, few-shot parameters, and semi-supervised iteration. The experimental hardware and software environments are the NVIDIA TITAN Xp with 12 GB memory and the Jupyter Notebook with libraries of Tensorflow (version 1.12.0), Numpy (version 1.19.2), Keras (version 2.2.4), and OpenCV (version 4.1).

### Comparison results with related work

In Ref [27], the few-shot plant diseases classification was carried out based on transfer learning and other optimized methods, such as contrastive loss and triplet loss. The used dataset was also PlantVillage, but the Ref [27] only considered one domain split. The first six classes are the target domain, and the rest 32 classes are the source domain. All the six classes in the target domain are tested, and k is 1, 5, 10, 15, 20, 30, 50, 80, and 100. In terms of the definition of N-way k-shot, the operation was called 6-way k-shot. Same with the above experimental settings, our semi-supervised few-shot results are compared with those provided in Ref [27], shown in Table 3.

**Table 3:** The comparison results with related work

Results	k-shot								
	1	5	10	15	20	30	50	80	100
Ref. [27]	0.56	0.72	0.77	0.8	0.82	0.86	0.88	0.9	0.91
Single SS	0.745	0.897	0.926	0.936	0.939	0.951	0.961	0.97	0.974
Iterative SS	0.751	0.9	0.927	0.936	0.939	0.951	0.961	0.97	0.974

In Table 3, the SS is short for the semi-supervised. It is shown that our proposed method outperforms the results presented in Ref [27] in all the conditions of k-shot. Specifically, the referred work achieved an average accuracy of 90% at 80-shot. However, our work achieved the average accuracy of 90% at 5-shot using the iterative semi-supervised, or 92.6% at 10-shot using the single semi-supervised method.

Thus, our semi-supervised methods have apparent advantages due to the reasonable use of the unlabeled samples.

### Further comparison experiments

In “Comparison results with related work” section, we compared the related work of the few-shot plant leaf diseases classification, which had shown our semi-supervised method's superiority. Since the different splits of the source domain and target domain will lead to the different fitness of transferred knowledge (network), we further carried out more comparison experiments considering domain split and few-shot parameters of N-way k-shot. The purpose of these comparison experiments is to verify the consistent correctness and generalization of our proposed semi-supervised method under different experimental settings.

According to the few-shot definition of N-way k-shot and the three different domain split modes described in Table 1, we set N as five and carried out each experiment ten times to obtain the average few-shot classification accuracy. The results under different domain splits are shown in Table 4.

**Table 4:** The comparison results under different domain splits

Results	Split-1, k-shot				Split-2, k-shot				Split-3, k-shot			
	1	5	10	20	1	5	10	20	1	5	10	20
Baseline	0.328	0.467	0.64	0.732	0.439	0.685	0.787	0.891	0.507	0.631	0.772	0.893
Single SS	0.337	0.509	0.667	0.747	0.447	0.747	0.857	0.897	0.523	0.676	0.799	0.901
Iterative SS	0.34	0.531	0.688	0.756	0.464	0.769	0.892	0.919	0.552	0.693	0.808	0.915

In Table 4, the SS is short for semi-supervised. There are three methods, named baseline, single semi-supervised, and iterative semi-supervised. The baseline stands for the typical few-shot classification based on transfer learning, shown in Fig. 4. The single SS and iterative SS methods are our proposed methods, shown in Figs. 5, 6.

Unlike the experimental settings in “Comparison results with related work” section, in this section, the N is equal to five, which means in each experiment, we randomly selected five classes from all the ten classes in the target domain to fine-tune the transferred model to distinguish these random N categories.

**Fig. 7:**
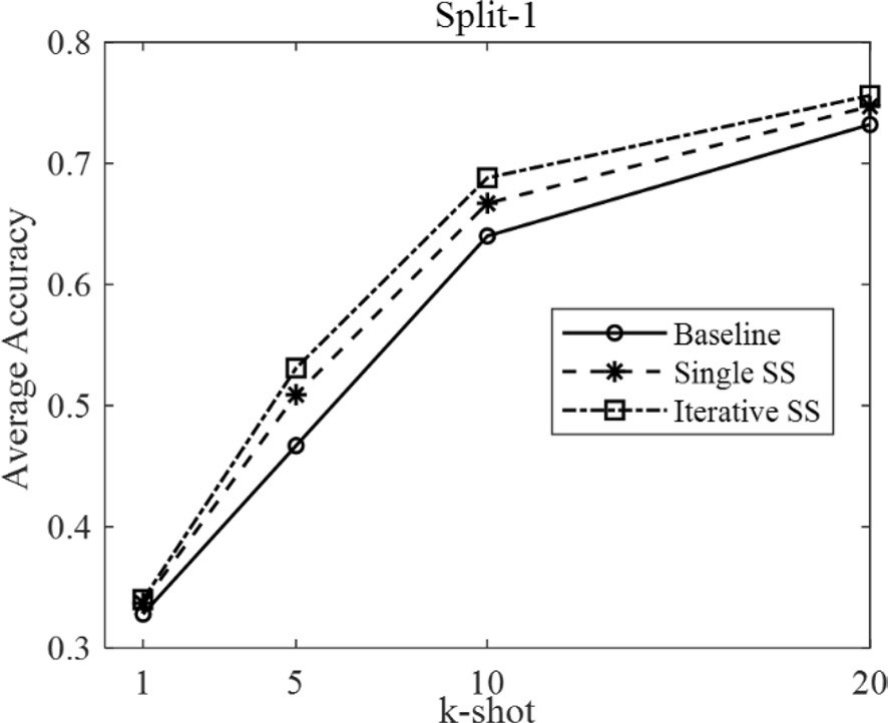
The average accuracy under domain split-1

**Fig. 8:**
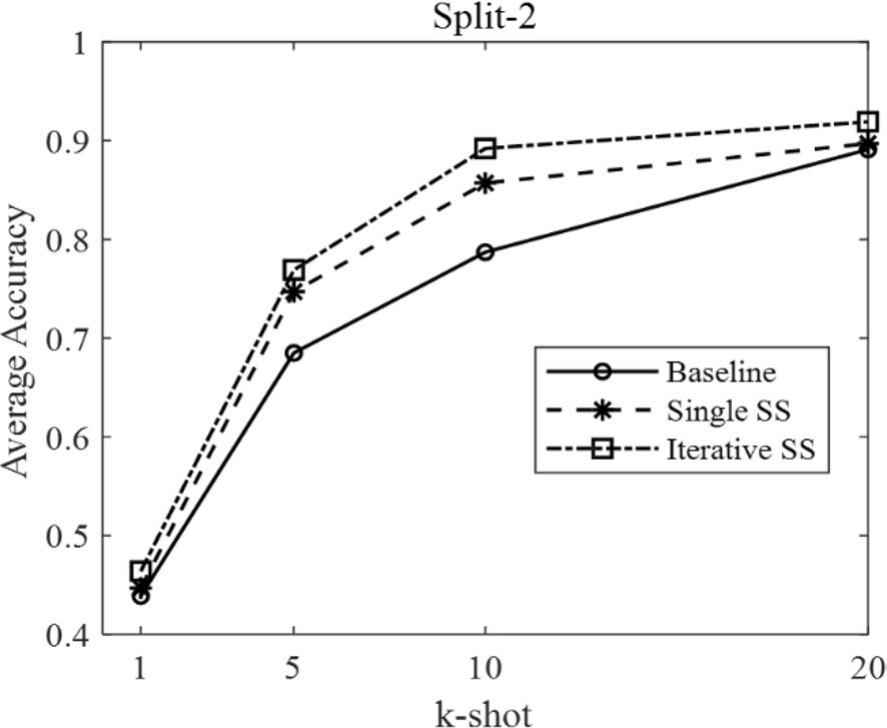
The average accuracy under domain split-2

**Fig. 9:**
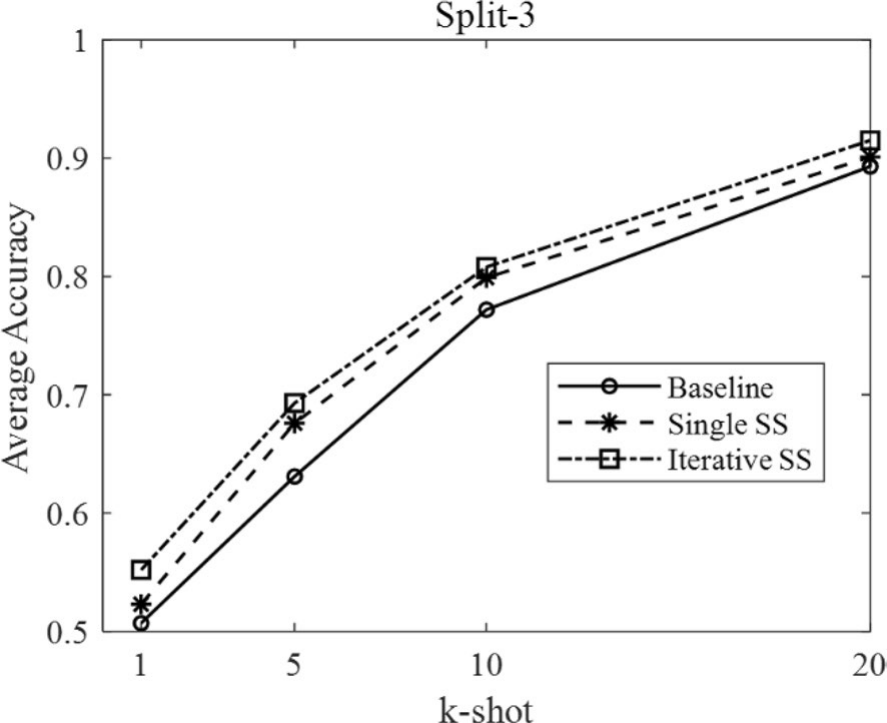
The average accuracy under domain split-3

The relation between average accuracy and k-shot is plotted in Figs. 7, 8, and 9, corresponding to the domain split-1, split-2, and split-3, respectively.

The few-shot parameters in Fig. 7 are as follows. The N-way is 5, and k-shot is 1, 5, 10, and 20. The data split mode is Split-1, which decides the different source domain and target domain.

The above three figures can intuitively indicate two points: First, the baselines are different under different domain splits, due to the different difficulty of few-shot task in different domain split modes. Second, under three different split modes, the proposed semi-supervised (SS) method is consistently higher than the baseline accuracy at each k-shot. In detail, the iterative SS method achieves the highest performance at the cost of more operations. Thus, the single SS method can be regarded as a suitable solution, balancing the performance gains with computational complexity.

Moreover, the average improvement by single SS method and iterative SS method on different k-shot can be calculated from Table 4. In particular, under the domain split-1, the average improvement by single SS method is 2.33%, and that by iterative SS method is 3.7%. Under the domain split-2, the average improvement by single SS method is 3.65%, and that by iterative SS method is 6.05%. Under the domain split-3, the average improvement by single SS method is 2.4%, and that by iterative SS method is 4.13%.

In summary, considering all the experimental factors, the average improvement by single SS method is 2.8%, and that by iterative SS method is 4.6%.

### Results of adaptive selection of pseudo-labeled samples

**Fig. 10:**
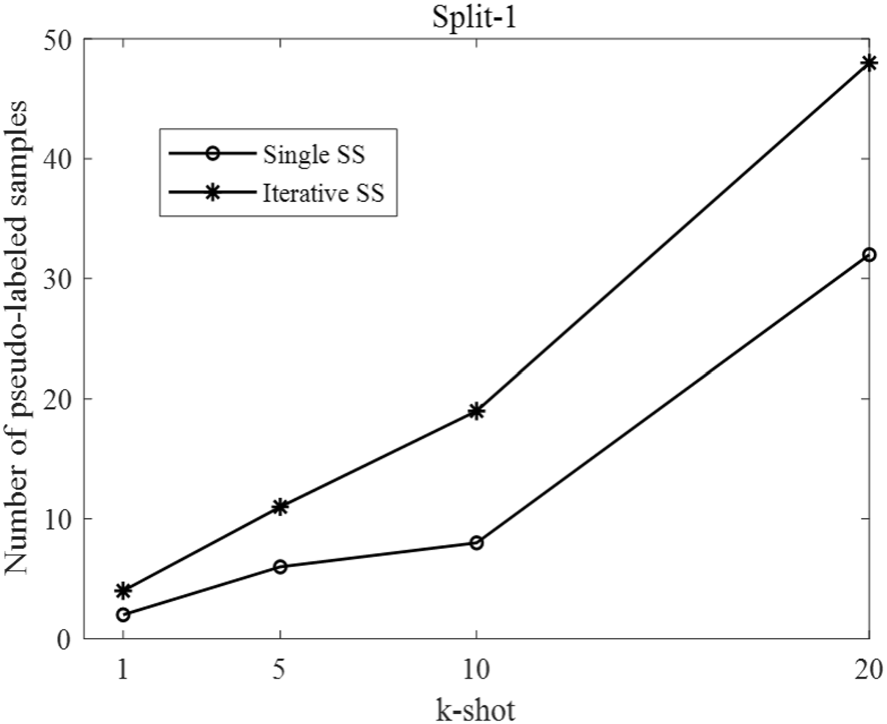
The number of pseudo-labeled samples under domain split-1

As described in “Adaptive selection of pseudo-labeled samples” section, pseudo-labeled samples' adaptive selection is crucial for the proposed semi-supervised few-shot methods. For instance, in the domain split-1, the number of adaptively selected pseudo-labeled samples under different k-shot is shown in Fig. 10.

It can be found that there is a positive correlation between the number of adaptively selected pseudo-labeled samples and k-shot. The reason is that with the increase of k-shot, the model has more training data to fine-tune. In other words, the model is stronger. So, it can be more confident to predict those unlabeled samples. When the predicted confidence is larger than 99.5%, the sample is selected for pseudo-labeling.

Besides, the iterative SS method will choose more pseudo-labeled samples than the single SS method. The reason is that the iterative SS method has one more fine-tuning stage than the single SS method. Thus, the model with iterative SS has better performance on understanding the tested categories to predict unlabeled data with higher confidence.

## Discussion

At present, the plant leaf diseases classification is mainly based on deep learning. Although there have been many achievements achieved, the drawbacks of deep learning cannot be ignored, e.g., the high cost of collecting and labeling large-scale datasets. As an essential supplement to deep learning, few-shot learning aims to combine a few samples and knowledge, committed to model learning and application deployment. The existing few-shot studies in the agricultural field all focus on the supervised paradigm and neglect the helpful information of unlabeled samples through the literature research. Thus, we want to explore the semi-supervised paradigm to improve the effect of few-shot classification and provide some inspirations for this community.

We proposed the single semi-supervised and iterative semi-supervised method to deal with few-shot plant leaf diseases classification. Overall, the experimental results are divided into two major sections. The first is the comparison with the related work in the Ref [27]. According to the experimental setting in referred work, the results have shown that our proposed method can outperform the reference method under all the conditions of k-shot. Specifically, the reference method achieved an average accuracy of 90% at 80-shot. Our work achieved an average accuracy of 90% at 5-shot with the iterative semi-supervised methods and 92.6% at 10-shot with the single semi-supervised method. In other words, under the same conditions, our methods can achieve better results with fewer samples. The second is the further comparison experiments considering more factors, e.g., the different three domain splits in Table 1 and different k-shot. The results consistently prove our semi-supervised methods can achieve better performance than the typical transfer-based few-shot learning. In detail, under the domain split-1, the average improvement by single SS method is 2.33%, and that by iterative SS method is 3.7%. Under the domain split-2, the average improvement by single SS method is 3.65%, and that by iterative SS method is 6.05%. Under the domain split-3, the average improvement by single SS method is 2.4%, and that by iterative SS method is 4.13%. Considering all the different domain splits and k-shot, the average improvement by single SS method is 2.8%, and that by iterative SS method is 4.6%.

This study did not consider some special case, such as the possible wrong data labels, which belongs to another important research scope of robustness. If the wrong labels are corresponding to original data, it is better to clean data first, otherwise they will mislead the learning process. If the wrong labels are corresponding to predicted data, it is suggested to modify the confidence interval to raise the screening criteria, and increase the number of iterations to improve model filtering performance.

In future work, from a broader and more practical perspective, we will try to do the few-shot classification under significant cross-domain by taking the public dataset as the source domain and the images taken in the field as the target domain. Moreover, in this study, the used model has seven convolution layers and two dense layers. As mentioned, the few-shot learning should aim at learning from few samples and convenient application deployment. Hence, we would like to further compress the size of the used model to realize the smaller intelligent model for convenient deployment.

## Conclusion

Automatic classification of plant leaf diseases based on a few labeled samples is significant to guarantee the yield and quality with low cost of data. In this work, we proposed the semi-supervised few-shot learning scheme, which can improve the average accuracy of few-shot classification by adaptively selecting the pseudo-labeled samples to help fine-tune the model. Through literature research, to our best knowledge, we carried out the first semi-supervised work in the field of few-shot plant diseases classification. The PlantVillage dataset was divided into three split modes, and extensive comparison experiments were executed to prove the correctness and generalization of proposed methods. Considering all the different domain splits and k-shot, the average improvement by the proposed single semi-supervised method is 2.8%, and that by the iterative semi-supervised method is 4.6%.

## Data Availability

The dataset used in this study is available in the Mendeley Data Repository, https://doi.org/10.17632/tywbtsjrjv.1.
